# Favorite Activity and Implications for Cognition, Mental Health, and Function in Persons With and Without Dementia

**DOI:** 10.1093/geroni/igab046.745

**Published:** 2021-12-17

**Authors:** Natalie Regier, Jeanine Parisi, Nancy Perrin, Laura Gitlin

**Affiliations:** 1 School of Nursing, Johns Hopkins School of Nursing, Maryland, United States; 2 Johns Hopkins Bloomberg School of Public Health, Baltimore, Maryland, United States; 3 Johns Hopkins University School of Nursing, Baltimore, Maryland, United States; 4 Drexel University, College of Nursing and Health Professions, Drexel University, Pennsylvania, United States

## Abstract

Little is known about the impact of engagement in personally meaningful activities for older adults. This study examines the impact of engagement in one’s favorite activity on cognitive, emotional, functional, and health-related outcomes in older adults with and without dementia. Data were obtained from 1,397 persons living with dementia (PLWD) and 4,719 cognitively healthy persons (CHP) participating in wave 2 of the National Health and Aging Trends Study (NHATS). Sociodemographic characteristics were examined by cognitive status. A multivariate analysis of variance indicated that, for PLWD, engagement in favorite activity was associated with greater functional independence and decreased depression (F(6,1201)=3.01, p<.01, Wilks’s Λ=.985, partial ƞ2=.015). For CHP, engagement in favorite activity was associated with greater functional independence, decreased depression and anxiety, and better performance on memory measures (F(6,4107)=11.46, p<.001, partial ƞ2=.016,). Findings suggest that engagement in personally meaningful activities may have significant and distinct benefits for persons with and without dementia.

